# Three-year outcome following neonatal encephalopathy in a high-survival cohort

**DOI:** 10.1038/s41598-022-12091-x

**Published:** 2022-05-13

**Authors:** Kennosuke Tsuda, Jun Shibasaki, Tetsuya Isayama, Akihito Takeuchi, Takeo Mukai, Yuichiro Sugiyama, Tomoaki Ioroi, Akihito Takahashi, Nanae Yutaka, Sachiko Iwata, Makoto Nabetani, Osuke Iwata

**Affiliations:** 1 Center for Human Development and Family Science, Department of Neonatology and Pediatrics, Nagoya City University Graduate School of Medical Sciences, Aichi, Nagoya 467-8601 Japan; 2 Department of Neonatology, Kanagawa Children’s Medical Center, Kanagawa, Japan; 3 Division of Neonatology, Center of Maternal-Fetal Neonatal and Reproductive Medicine, National Center for Child Health and Development, Tokyo, Japan; 4 Division of Neonatology, National Hospital Organization Okayama Medical Center, Okayama, Japan; 5 Department of Pediatrics, The University of Tokyo Hospital, Tokyo, Japan; 6 Department of Pediatrics, Japanese Red Cross Aichi Medical Center Nagoya Daiichi Hospital, Aichi, Japan; 7 Department of Pediatrics, Perinatal Medical Center, Himeji Red Cross Hospital, Hyogo, Japan; 8 Department of Pediatrics, Kurashiki Central Hospital, Okayama, Japan; 9 Department of Pediatrics, Yodogawa Christian Hospital, Osaka, Japan

**Keywords:** Neonatal brain damage, Neonatology

## Abstract

This study investigated the 3-year clinical outcomes in relation to the severity of encephalopathy in high-survival infants who underwent therapeutic hypothermia. This retrospective observational study was conducted in level II/III neonatal intensive care units in Japan. The nationwide cohort included 474 infants registered in the Baby Cooling Registry of Japan between January 2012 and December 2016. Clinical characteristics, mortality rate and severe neurological impairment at age 3 years were evaluated. Of the infants, 48 (10.4%), 291 (63.1%) and 122 (26.5%) had mild, moderate and severe encephalopathy, respectively, upon admission. By age 3, 53 (11.2%) infants died, whereas 110 (26.1%) developed major disabilities. The mild group survived up to age 3. In the moderate group, 13 (4.5%) died and 44 (15.8%) developed major disabilities. In the severe group, 39 (32.0%) died by age 3. Adverse outcomes were observed in 100 (82.0%) infants. Mortality was relatively low in all subgroups, but the incidence of major disabilities was relatively high in the severe group. The relatively low mortality and high morbidity may be due to Japanese social and ethical norms, which rarely encourage the withdrawal of intensive life support. Cultural and ethical backgrounds may need to be considered when assessing the effect of therapeutic interventions.

## Introduction

Hypoxic-ischaemic encephalopathy (HIE) contributes to both infant mortality and long-term morbidity in childhood^1^. The prevalence of moderate or severe HIE is 0.4–2.0 cases per 1000 births in high-income countries, including Japan, United States and European countries^2–4^. Therapeutic hypothermia is the only neuroprotective treatment that has been proven to reduce death and neurological impairments in infants with moderate or severe HIE^5^. However, the outcomes of HIE in infants are diverse, even after therapeutic hypothermia. In early large-scale randomised controlled trials of therapeutic hypothermia, the mortality rate and composite outcome of death or moderate to severe neurological impairments among cooled infants by 2 years of age were 23.1–33.3% and 39.2–56.0%, respectively^5^. A more recent randomised controlled trial, which provided therapeutic hypothermia (32.0 °C or 33.5 °C for either 72 or 120 h) to encephalopathic infants, reported relatively lower mortality rates (8.7–19.4%) and adverse composite outcomes (29.3–34.5%)^6^. A lower mortality rate (7.5%) and similar composite adverse outcome rate (29.5%) were reported in a study assessing 18-month follow-up data from the Japanese national registry^7^. Furthermore, a subgroup analysis of infants with a 10-min Apgar score of zero from the same registry indicated a relatively low mortality rate (32% versus 48–54% in previous studies), as well as a high incidence of moderate to severe neurological impairments among survivors (84% versus 42–55% in previous studies)^8–11^. The relatively low mortality and high morbidity rates observed in the survivors were attributed to the cultural and ethical norms in Japan, where physicians rarely propose the withdrawal of intensive life support, even for infants with the most severe degrees of HIE^11^.

By contrast, mild HIE has rarely been a focus of study, as its prognosis is generally considered favourable^5,12–14^. However, a recent study of 43 infants found that 16% of uncooled infants with mild HIE had neurological impairments by age 18–22 months^15^. Despite the lack of clinical evidence to support the benefit of therapeutic hypothermia for infants with mild HIE, a considerable proportion of these infants have received such treatment in Western countries^16,17^. However, such a ‘therapeutic creep’ has not been as apparent in Japan, possibly due to a nationwide dissemination campaign encouraging physicians to adhere to evidence-based therapeutic hypothermia regimens^18^.

Thus, for an appraisal of preferred neuroprotective regimens, domestic backgrounds, such as cultural and ethical backgrounds and local adherence to evidence-based guidelines, may need to be considered. To facilitate comparisons of the benefits of cooling among studies conducted in different countries, clinical outcomes need to be assessed in conjunction with the level of HIE severity.

Thus, this study aimed to review the 3-year mortality rate and incidence of severe neurodevelopmental impairments according to the initial HIE severity in a Japanese cohort of infants with a high survival rate following therapeutic hypothermia.

## Results

### Final study cohort

**Figure 1:**
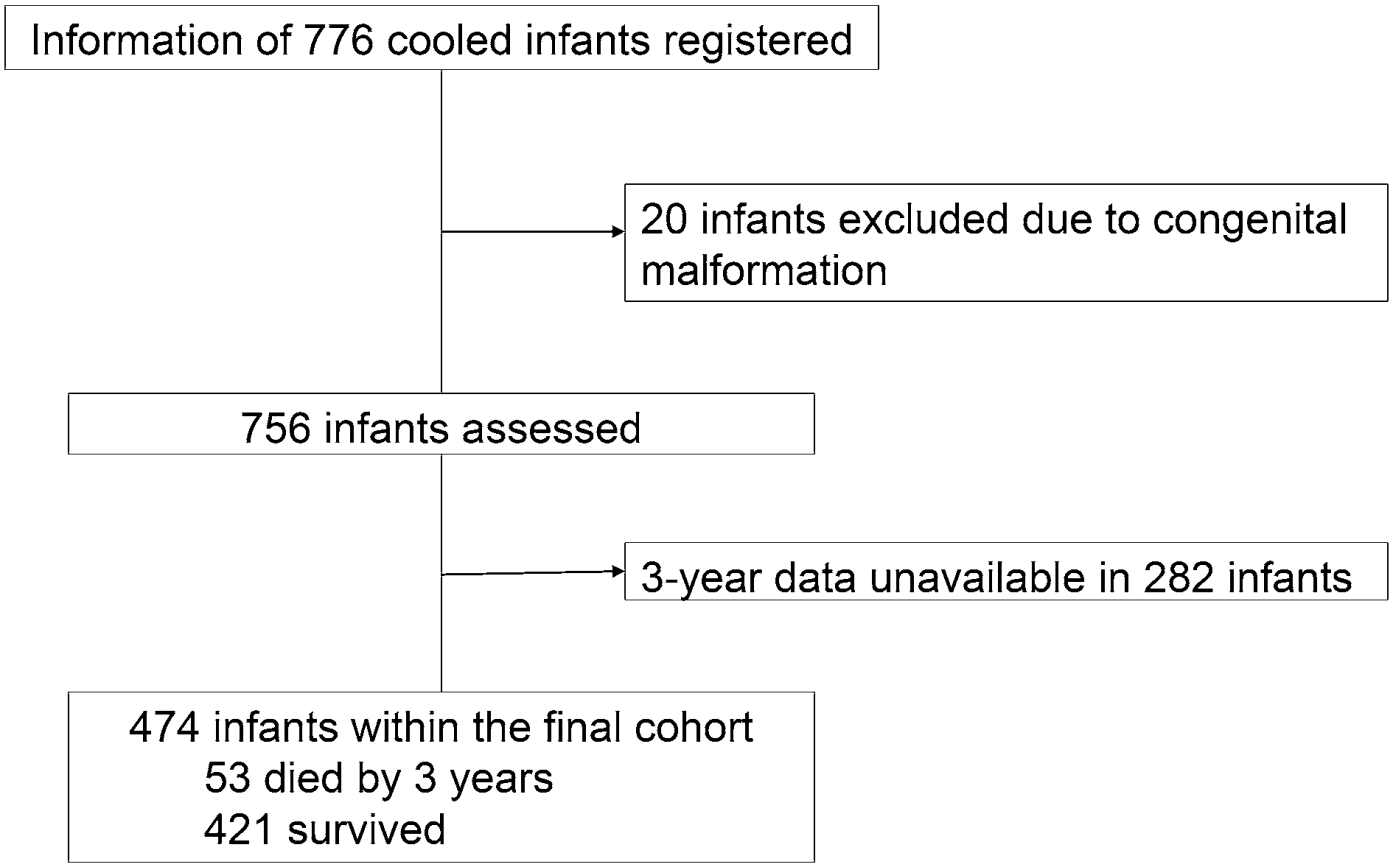
Profile of the study population. A diagram depicting the flow of the study population.

Clinical data were obtained from 756 infants, who were registered at 110 intensive care units over the 5-year window period. Among these infants, 474 (62.7%, final cohort) had outcome data at 3 years of age; 282 were lost to follow-up (Fig. 1). The baseline characteristics were similar between those with and without follow-up data; an exception to this was that the encephalopathy stage was more severe among infants in the final study cohort (Table 1).

**Table 1:** Baseline characteristics of infants with or without confirmed outcomes.

Variables	Outcome information		P
	Available (n = 474)	Not available (n = 282)	
**Background variables**			
Gestational age (weeks)	38.5 ± 1.7	38.6 ± 1.7	0.445
Birth weight (g)	2869 ± 485	2918 ± 515	0.197
**Birth location**			
Inborn	140 (30.0)	76 (27.6)	
Outborn	327 (70.0)	199 (72.4)	0.498
10-min Apgar score	5 [3–7]	5 [4–7]	0.227
First blood gas pH	6.94 ± 0.21	6.95 ± 0.21	0.650
Base deficit (mmol/L)	14.4 ± 10.4	14.4 ± 10.4	0.994
Thompson score at admission	11 [9–15]	11 [8–14]	0.066
24 h after initiating cooling	11 [8–14]	11 [7–14]	0.392
**Sarnat stage at admission**			
Mild	48 (10.4)	45 (16.7)	
Moderate	291 (63.1)	172 (63.7)	
Severe	122 (26.5)	53 (19.6)	0.014

Values are shown as the number (%), mean ± standard deviation or median [interquartile range]. P-values were determined via Student’s t-test (unpaired, two-tailed), Mann–Whitney U-test or Chi-square test, without correction for multiple comparisons.

### Encephalopathy stages and clinical course during hospitalisation

Among 474 infants treated with therapeutic hypothermia, encephalopathy at admission was mild in 48 (10.4%) infants, moderate in 291 (63.1%) and severe in 122 (26.5%); data were unavailable for 13 infants. The rate of emergency delivery, gestational age and body weight at birth were similar by the severity of HIE. In contrast, there was a trend that greater HIE severities were associated with being outborn, low Apgar scores at 10 min, need for resuscitation beyond 10 min and more severe metabolic acidosis represented by lower blood pH and greater base deficit (see online Supplementary Table for details and other clinical backgrounds, including the Thompson encephalopathy scores, presence of seizures and requirement for tube feeding and mechanical ventilation).

### Outcomes at 3 years of age

Among the 474 infants in the final cohort, 53 (11.2%) died by the time of the 3-year follow-up, whereas 110 (26.1%) survivors developed major disabilities; 163 (34.4%) cases of death or major disability were recorded (Table 2). Among the surviving children, 67 (15.9%) and 44 (10.5%) were dependent on tube feeding and respiratory support, respectively. Hearing loss, blindness, epilepsy, Gross Motor Function Classification System^19^ (GMFCS) > 2 and Manual Ability Classification System^20^ (MACS) > 2 were noted in 28 (6.7%), 12 (2.9%), 14 (3.3%), 89 (21.1%) and 97 (23.0%) children, respectively.

**Table 2:** Outcomes of cooled infants at 3 years of age.

Outcomes	All n = 474	Sarnat staging at admission		
		Mild n = 48	Moderate n = 291	Severe n = 122
**Primary outcome**				
Death or major disability^a^	163 (34.4)	2 (4.2)	57 (19.6)	100 (82.0)
**Secondary outcomes**				
Death	53 (11.2)	0 (0.0)	13 (4.5)	39 (32.0)
Survival	421 (88.8)	48 (100.0)	278 (95.5)	83 (68.0)
Major disability^a^	110 (26.1)	2 (4.2)	44 (15.8)	61 (73.5)
**Dependence on medical support**				
Tube feeding	67 (15.9)	0 (0.0)	20 (7.2)	45 (54.2)
Respiratory support	44 (10.5)	0 (0.0)	12 (4.3)	31 (37.3)
Any of the above	69 (16.4)	0 (0.0)	21 (7.6)	46 (55.4)
**Sensory impairment**				
Hearing loss	28 (6.7)	0 (0.0)	10 (3.6)	17 (20.5)
Blindness	12 (2.9)	0 (0.0)	5 (1.8)	7 (8.4)
Epilepsy	14 (3.3)	1 (2.1)	5 (1.8)	8 (9.6)
**Gross Motor Function Classification System**				
Levels 3–5	89 (21.1)	2 (4.2)	30 (10.8)	55 (66.3)
**Manual Ability Classification System**				
Levels 3–5	97 (23.0)	2 (4.2)	35 (12.6)	58 (69.9)

Values are shown as the number (%).

Percentages are based on the number of infants for whom data were available.

^a^Defined as survival with at least one of the following conditions: requirement for tube feeding or respiratory support, hearing loss, blindness, epilepsy, Gross Motor Function Classification System and Manual Ability Classification System scores > 2.

### Encephalopathy stages and outcomes

All 48 infants diagnosed with mild encephalopathy upon admission survived to 3 years of age without hearing loss, blindness or requirement for chronic healthcare. Of the infants with mild encephalopathy, 4.2% (2/48) had a major disability; one infant was diagnosed with cerebral palsy (GMFCS level 3), while another infant was diagnosed with both cerebral palsy (GMFCS level 5) and epilepsy (Table 2).

Of the 291 infants diagnosed with moderate encephalopathy upon admission, 13 (4.5%) died, while 44 (15.8%) survivors developed a major disability; 57 (19.6%) cases of composite adverse outcomes were recorded. Among survivors, 21 (7.6%) were dependent on respiratory support or tube feeding. At 3 years of age, 10 (3.6%), 5 (1.8%), 5 (1.8%), 30 (10.8%) and 35 (12.6%) children exhibited hearing loss, blindness, epilepsy, GMFCS > 2 and MACS > 2, respectively (Table 2).

Of the 122 infants with severe encephalopathy, 39 (32.0%) died by 3 years of age; 100 (82.0%) cases of death or major disability were noted. Among the survivors, 46 (55.4%) children required respiratory support or tube feeding. Hearing loss, blindness, epilepsy, GMFCS > 2 and MACS > 2 were noted in 17 (20.5%), 7 (8.4%), 8 (9.6%), 55 (66.3%) and 58 (69.9%) children, respectively, at 3 years of age (Table 2).

## Discussion

In a cohort of infants with high-survival rate from the Japanese national registry, the relationship between the severity of encephalopathy and outcomes following therapeutic hypothermia differed from that reported in other developed countries^5,21^. The 3-year outcomes of Japanese HIE infants were characterised by consistently low mortality rates for those diagnosed with mild, moderate and severe encephalopathy (0.0%, 4.5% and 32.0%, respectively), as well as a relatively high rate of major disabilities in survivors of severe encephalopathy (73.5%). The social and ethical norms in Japan, which do not allow the withdrawal of intensive life support (despite severe neurological impairment) may have accounted for the low mortality and high morbidity rates observed among infants with severe encephalopathy.

A meta-analysis based on clinical studies from Western countries reported mortality rates of 13.5% and 52.4% in infants with moderate and severe neonatal encephalopathy, respectively, following therapeutic hypothermia^5^; these rates were considerably higher than the mortality rates documented among Japanese infants in the present study (4.5% and 32.0%, respectively). Another study utilising an international registry in The Children’s Hospitals Neonatal Database (n = 945) reported hospital mortality rates of 3.5% and 46.8% for infants with moderate and severe encephalopathy, respectively. The majority of deaths (82.4% and 86.1% of deaths following moderate and severe encephalopathy, respectively) occurred after the withdrawal of life-sustaining support^21^. Although our current study did not collect information regarding withdrawal of intensive life support, our prior study showed that even among infants with most severe encephalopathy (with 10-min Apgar scores of zero), only 2 of the 9 (22.2%) died following withdrawal^11^. In the current study, the rate of major disability among survivors of severe encephalopathy was 73.5%, which was relatively higher than the morbidity rate (36.8%) reported in a previous study^5^. The association of severe encephalopathy with the combination of low mortality (32%) and high morbidity (57%, severe disabilities) was also observed in a previous study conducted among severely asphyxiated infants (10-min Apgar scores of zero) in Japan^11^. These findings may be attributed to the reluctance among physicians in Japan to withdraw intensive life support, even in the most severe cases of encephalopathy. Future studies of neuroprotective treatments may need to consider the influence of local social and ethical backgrounds to more accurately assess the effect of therapeutic hypothermia on long-term morbidity and mortality rates.

To date, only a few studies have addressed the outcomes of infants with mild HIE at or beyond 18 months of age^22,23^. However, recent studies have reported that these infants are at a risk of adverse outcomes, such as behavioural problems and neurodevelopmental impairments^24,25^. For example, a multicentre cohort study demonstrated that 16% of infants with mild encephalopathy who did not undergo therapeutic hypothermia had cognitive developmental quotients of < 85, while approximately 40% had at least one cognitive, motor or language score of < 85^15^. In the present study, only 4.2% of infants with mild encephalopathy developed adverse outcomes at 3 years of age. In this study, the majority of the registered infants had moderate to severe HIE; this is perhaps due to the Baby Cooling Registry of Japan specifically recruiting infants who underwent therapeutic hypothermia and the strict adherence to international guidelines in Japan, where therapeutic hypothermia is considered only in cases with moderate to severe HIE^18^. It is possible that the HIE severity in the 48 patients with mild HIE in this study was different from that in previous studies. Although both the mortality rate and long-term outcome of these infants were optimal, the influence of therapeutic hypothermia remains unclear. Further studies are required to determine whether therapeutic hypothermia is beneficial in this cohort of infants.

We were able to elucidate the relationship between the severity of encephalopathy, as assessed in infants upon admission, and long-term outcomes. This was achieved by assessing a high-survival cohort from a large registry in Japan. However, several limitations should be considered. First, 37.3% of the infants were lost to follow-up. The follow-up rate was lower in infants with mild encephalopathy, presumably reflecting the optimistic perspective of the neurological outcome and subsequent cessation of follow-up. Therefore, our current findings may predominantly reflect outcomes associated with infants with relatively more severe disease. Second, as the assessment of neurological outcomes using individualised batteries was only performed in 40% of the study population, the use of standard outcome measures was problematic. Similarly, we were unable to incorporate potential independent variables of outcomes within the analysis, such as the type of respiratory care, use of inotropic support, incidence of pulmonary hypertension and seizures and findings of amplitude-integrated electroencephalogram (aEEG) and MRI, because the type, dose and duration of supportive treatment and assessment of ultrasound sonography, aEEG and MRI significantly differ between participating units^26^. The evaluation of outcomes focused on chronic healthcare needs and motor function, but not on cognitive or language function, as reported in previous studies^27–29^. Third, although the Baby Cooling Registry of Japan disseminated tools and domestic guidelines for the evidence-based implementation of therapeutic hypothermia, including the precise assessment of encephalopathy using the Sarnat encephalopathy staging^12^ and the Thompson encephalopathy scoring^30^, the classification of the severity of encephalopathy was not standardised for this particular registry across centres. Recently, Chalak et al. demonstrated that the long-term outcomes of encephalopathic infants can be predicted using a relatively more objective categorisation of the modified Sarnat scoring criteria^31^. This may improve patient selection in future prospective studies.

In conclusion, the incidence of death and major disability following mild, moderate and severe HIE was reviewed for a large-scale national cohort in Japan. Mortality was relatively low in all subgroups (patients were stratified based on HIE severity), whereas the incidence of major disabilities was relatively high in infants with severe HIE. Cultural and ethical backgrounds, as well as the quality of medical care, may need to be considered when assessing the benefits of therapeutic interventions for HIE. Although the outcomes of infants with mild HIE appeared to be optimal following therapeutic hypothermia, the benefit of such treatment for this cohort of infants needs to be validated further. The natural outcomes associated with mild HIE and the neuroprotective effect of therapeutic interventions need to be addressed in future studies.

## Methods

### Population and data collection

The Baby Cooling Registry of Japan is an online case registry that was established in January 2012 and includes patient data from all registered Japanese level II/III neonatal intensive care centres. The details of this registry have been reported previously^18^. This observational study was based on 3-year outcome data from 474 infants registered between January 1, 2012, and December 31, 2016 (Fig. 1).

### Assessment of outcomes

The outcomes of cooled infants were assessed at 3 years of age according to the protocol of the Baby Cooling Registry of Japan^18^. The infants’ parents were asked to assess the outcome of their cooled infants at 18 months postconceptional age and 36 months chronological age by consulting a neonatologist, paediatrician or child neurologist. Data pertaining to the following variables were collected: hearing loss; blindness; epilepsy (requiring anticonvulsant treatment); chronic healthcare needs (e.g., requirements for tube feeding, gastrostomy, tracheotomy or prolonged ventilator management upon reaching the age of 3 years old); and neuromotor function assessed using the GMFCS^19^ and MACS^20^. GMFCS and MACS scores range from 0 to 5, with higher scores indicating greater impairment. Major disability was defined as survival with at least one of the following conditions: requirement for tube feeding or respiratory support, hearing loss, blindness, epilepsy, GMFCS > 2 or MACS > 2.

### Statistical analysis

The primary outcome was death or major disability upon the 3rd year of follow-up. To assess potential bias due to follow-up loss, baseline characteristics were compared between children with and without follow-up data at 3 years of age using Student’s t-test, Mann–Whitney U test or Chi-squared test, where appropriate. Statistical findings were not corrected for multiple comparisons. For all analyses, the level of significance was set at P < 0.05. Values are shown as a number (proportion, %) for categorical variables or mean ± standard deviation (or median and interquartile range) for quantitative variables.

### Ethics approval and consent

This study was conducted in accordance with the principles of the Declaration of Helsinki. The registry protocols were approved by the Ethics Committees of the Kurume University School of Medicine and Saitama Medical University, Japan. The Ethics Committee of Kurume University School of Medicine approved that informed consent was not required because only anonymised data obtained for clinical reasons were used in the study.

## Supplementary Information

Supplementary Information.

## Data Availability

The datasets generated during and/or analysed during the current study are available from the corresponding author on reasonable request.

## References

[1] Lawn JE (2014). Every Newborn: Progress, priorities, and potential beyond survival. Lancet.

[2] Hayakawa M (2014). Incidence and prediction of outcome in hypoxic–ischemic encephalopathy in Japan. Pediatr. Int..

[3] Kurinczuk JJ, White-Koning M, Badawi N (2010). Epidemiology of neonatal encephalopathy and hypoxic–ischaemic encephalopathy. Early Hum. Dev..

[4] Lee AC (2013). Intrapartum-related neonatal encephalopathy incidence and impairment at regional and global levels for 2010 with trends from 1990. Pediatr. Res..

[5] Jacobs SE (2013). Cooling for newborns with hypoxic ischaemic encephalopathy. Cochrane Database Syst. Rev..

[6] Shankaran S (2017). Effect of depth and duration of cooling on death or disability at age 18 months among neonates with hypoxic–ischemic encephalopathy: A randomized clinical trial. JAMA.

[7] Tsuda K (2021). Body temperature, heart rate and long-term outcome of cooled infants: An observational study. Pediatr. Res..

[8] Kasdorf E, Laptook A, Azzopardi D, Jacobs S, Perlman JM (2015). Improving infant outcome with a 10 min Apgar of 0. Arch. Dis. Child Fetal Neonatal Ed..

[9] Natarajan G (2013). Apgar scores at 10 min and outcomes at 6–7 years following hypoxic–ischaemic encephalopathy. Arch. Dis. Child Fetal Neonatal Ed..

[10] Laptook AR (2009). Outcome of term infants using apgar scores at 10 minutes following hypoxic–ischemic encephalopathy. Pediatrics.

[11] Shibasaki J (2020). Outcomes related to 10-min Apgar scores of zero in Japan. Arch. Dis. Child Fetal Neonatal Ed..

[12] Sarnat HB, Sarnat MS (1976). Neonatal encephalopathy following fetal distress. A clinical and electroencephalographic study. Arch. Neurol..

[13] Robertson CM, Finer NN, Grace MG (1989). School performance of survivors of neonatal encephalopathy associated with birth asphyxia at term. J. Pediatr..

[14] Handley-Derry M (1997). Intrapartum fetal asphyxia and the occurrence of minor deficits in 4- to 8-year-old children. Dev. Med. Child Neurol..

[15] Chalak LF (2018). Prospective research in infants with mild encephalopathy identified in the first six hours of life: Neurodevelopmental outcomes at 18–22 months. Pediatr. Res..

[16] Mehta S (2017). Eligibility criteria for therapeutic hypothermia: From trials to clinical practice. J. Paediatr. Child Health.

[17] Oliveira V (2018). Therapeutic hypothermia in mild neonatal encephalopathy: A national survey of practice in the UK. Arch. Dis. Child Fetal Neonatal Ed..

[18] Tsuda K (2017). Therapeutic hypothermia for neonatal encephalopathy: A report from the first 3 years of the Baby Cooling Registry of Japan. Sci. Rep..

[19] Palisano R (1997). Development and reliability of a system to classify gross motor function in children with cerebral palsy. Dev. Med. Child Neurol..

[20] Eliasson AC (2006). The Manual Ability Classification System (MACS) for children with cerebral palsy: Scale development and evidence of validity and reliability. Dev. Med. Child Neurol..

[21] Massaro AN (2015). Short-term outcomes after perinatal hypoxic ischemic encephalopathy: A report from the Children's Hospitals Neonatal Consortium HIE focus group. J. Perinatol..

[22] Murray DM, O'Connor CM, Ryan CA, Korotchikova I, Boylan GB (2016). Early EEG grade and outcome at 5 years after mild neonatal hypoxic ischemic encephalopathy. Pediatrics.

[23] van Kooij BJ (2010). Serial MRI and neurodevelopmental outcome in 9- to 10-year-old children with neonatal encephalopathy. J. Pediatr..

[24] Finder M (2020). Two-year neurodevelopmental outcomes after mild hypoxic ischemic encephalopathy in the era of therapeutic hypothermia. JAMA Pediatr..

[25] Conway JM, Walsh BH, Boylan GB, Murray DM (2018). Mild hypoxic ischaemic encephalopathy and long term neurodevelopmental outcome—A systematic review. Early Hum. Dev..

[26] Iwata O (2012). Hypothermia for neonatal encephalopathy: Nationwide Survey of Clinical Practice in Japan as of August 2010. Acta Paediatr..

[27] Guillet R (2012). Seven- to eight-year follow-up of the CoolCap trial of head cooling for neonatal encephalopathy. Pediatr. Res..

[28] Azzopardi D (2014). Effects of hypothermia for perinatal asphyxia on childhood outcomes. N. Engl. J. Med..

[29] Shankaran S (2012). Childhood outcomes after hypothermia for neonatal encephalopathy. N. Engl. J. Med..

[30] Thompson CM (1997). The value of a scoring system for hypoxic ischaemic encephalopathy in predicting neurodevelopmental outcome. Acta Paediatr..

[31] Chalak LF, Adams-Huet B, Sant'Anna G (2019). A total sarnat score in mild hypoxic–ischemic encephalopathy can detect infants at higher risk of disability. J. Pediatr..

