# Supplementary material for: Three-year outcome following neonatal encephalopathy in a high-survival cohort
Source: Sci Rep. 2022 May 13;12:7945. doi: 10.1038/s41598-022-12091-x (PMC9106703; doi:10.1038/s41598-022-12091-x)
Supplement: Supplementary file 1 — Supplementary Information. [file 41598_2022_12091_MOESM1_ESM.docx]

**Supplementary File**

**TITLE**

Three-year outcome following neonatal encephalopathy in a high-survival cohort

**AUTHORS**

Kennosuke Tsuda^1*^, Jun Shibasaki^2^, Tetsuya Isayama^3^, Akihito Takeuchi^4^, Takeo Mukai^5^, Yuichiro Sugiyama^6^, Tomoaki Ioroi^7^, Akihito Takahashi^8^, Nanae Yutaka^9^, Sachiko Iwata^1^, Makoto Nabetani^9^, Osuke Iwata^1^.

**AFFILIATIONS**

1. Center for Human Development and Family Science, Department of Neonatology and Pediatrics, Nagoya City University Graduate School of Medical Sciences, Aichi, Japan
2. Department of Neonatology, Kanagawa Children's Medical Center, Kanagawa, Japan
3. Division of Neonatology, Center of Maternal-Fetal Neonatal and Reproductive Medicine, National Center for Child Health and Development, Tokyo, Japan
4. Division of Neonatology, National Hospital Organization Okayama Medical Center, Okayama, Japan
5. Department of Pediatrics, The University of Tokyo Hospital, Tokyo, Japan
6. Department of Pediatrics, Japanese Red Cross Aichi Medical Center Nagoya Daiichi Hospital, Aichi, Japan
7. Department of Pediatrics, Perinatal Medical Center, Himeji Red Cross Hospital, Hyogo, Japan
8. Department of Pediatrics, Kurashiki Central Hospital, Okayama, Japan
9. Department of Pediatrics, Yodogawa Christian Hospital, Osaka, Japan

**CORRESPONDING AUTHOR:**

Dr Kennosuke Tsuda, Center for Human Development and Family Science, Department of Pediatrics and Neonatology, Nagoya City University Graduate School of Medical Sciences, Aichi, Nagoya 467-8601, Japan; [kentsuda@med.nagoya-cu.ac.jp](mailto:kentsuda@med.nagoya-cu.ac.jp)

**Online Supplemental Table: Clinical details of the included infants**

|  |  | Sarnat staging at admission | | |
| --- | --- | --- | --- | --- |
| **Variables** | All  n = 474 | Mild  n = 48 | Moderate  n = 291 | Severe  n = 122 |
| Gestational age (weeks) | 38.5 ± 1.7 | 38.3 ± 2.0 | 38.5 ± 1.8 | 38.5 ± 1.7 |
| Birth weight (g) | 2869 ± 485 | 2785 ± 486 | 2874 ± 473 | 2896 ± 504 |
| Outborn | 327 (70.0) | 25 (52.1) | 208 (71.5) | 91 (74.6) |
| Emergency delivery* | 342 (73.5) | 35 (72.9) | 204 (71.1) | 98 (80.3) |
| 10-minute Apgar score | 5 [3–7] | 7 [5–8] | 5 [4–7] | 2 [1–4] |
| Need for resuscitation > 10 minutes | 377 (87.1) | 20 (52.6) | 238 (89.1) | 112 (93.3) |
| First blood gas pH | 6.94 ± 0.21 | 6.95 ± 0.16 | 6.97 ± 0.20 | 6.85 ± 0.22 |
| Base deficit (mmol/L) | 14.4 ± 10.4 | 12.4 ± 8.9 | 12.7 ± 9.7 | 19.7 ± 10.7 |
| Thompson score at admission | 11 [9–15] | 6.5 [4–10] | 10 [8–12] | 16 [15–17] |
| 24 h after initiating cooling | 11 [8–14] | 8 [5–12] | 10 [7–12] | 16 [12–18] |
| Seizure before cooling | 145 (32.4) | 4 (8.7) | 80 (28.5) | 60 (50.0) |
| Duration of tube feeding (days) | 11.5 [9–15] | 9.5 [8–15] | 11 [9–14] | 17.5 [12–30] |
| Duration of mechanical ventilation (days) | 6 [5–8] | 6 [5–7] | 6 [5–7] | 9 [7–15] |

Values are shown as the number (%), mean ± standard deviation or median [interquartile range].

*Including emergency caesarean, forceps and vacuum-assisted vaginal delivery.
